# Speech recognition thresholds in noisy areas: Reference values for normal hearing adults

**DOI:** 10.1016/S1808-8694(15)31087-9

**Published:** 2015-10-19

**Authors:** Marília Oliveira Henriques, Elisiane Crestani de Miranda, Maristela Julio Costa

**Affiliations:** 1 Master in Human Communication Disorders. Speech and Hearing Therapist; 2 Speech and hearing therapist; 3 PhD in Human Communication Disorders. Adjunct Professor - Speech and hearing therapy program - Federal University of Santa Maria. Universidade Federal de Santa Maria

**Keywords:** audiology, noise, tests of speech discrimination

## Abstract

In audiology clinics, complaints about difficulties in speech recognition in noise environments are frequent, even for normal-hearing individuals. Thus, the audiologist must not only identify a hearing loss, but also analyze speech recognition, under noisy conditions similar to those found in our daily lives.

**Aim:**

Determine the reference value for the recognition of phrases under noisy conditions, in the free field, for adult normal hearing patients.

**Materials and Methods:**

This study was carried out in 2005 and 2006. We had 150 adult normal hearing individuals participating, with ages between 18 and 64 years, assessed in a soundproof booth. We evaluation was based on lists of phrases in Portuguese. The phrases lists were presented in the free field, in the presence of a competitive noise, at the fixed intensity of 65 dB H. The incidence angle of both stimuli was 0°- 0° azimuth.

**Results and Conclusion:**

The phrases recognition thresholds in the free fields were obtained in the signal to noise ratio of −8.14 dB H, which is the reference value for normal hearing individuals.

## INTRODUCTION

In order to have an efficient oral communication process, three elements are necessary: speaker, message, and listener. However, message understanding is sometimes impaired by competitive noise.

Nonetheless, in audiology clinics, complaints of difficulty to understand speech in noisy environments are increasingly more frequent, even in individuals considered audiologically normal, from the qualitative standpoint. For this reason, it is fundamental to be able to analyze the relationship between audiometric thresholds and the capacity to recognize speech in the process of audiologic evaluation.

In order to measure the hearing difficulties of the patient under evaluation, the audiologist must use a number of tests that not only will allow for the identification of hearing loss, but will also analyze the understanding of auditory stimuli, including speech, in the clinical setting and, especially, under conditions similar to the ones found in our daily lives.

Despite the growing concern with this reality in recent years, the interest in understanding and solving problems involving speech intelligibility, especially under noisy situations, started many decades ago. During World War II, the first tests with such goal were created, with the creation of hearing rehabilitation programs for soldiers who came back from the war with hearing impairment. In 1956 some researchers^1^ reported that one of the main limitations of the tests that used pure tone as stimulus is the fact that they do not assess the so called “social hearing” of the individual. With that, they emphasized the need to use tests with speech stimuli for such end.

Since then, in different countries, these tests are being developed, studied and enhanced in order to be routinely used in the clinical setting. Some of them use mono or dissyllabic words as stimuli. However, the use of entire phrases is an important option, because they represent the means of assessment which is closer to in daily life situations. Some authors^2^, ^3^, ^4^, ^5^, ^6^, ^7^, besides using phrases as a stimulus, developed the equivalent noise together with the speech material, in order to assess speech recognition in silence and also in face of a competitive noise. In Brazil, the Lists of Phrases in Portuguese (LPP)^8^ was pioneer in this field.

Different application strategies have been suggested for these tests. One of them is to present speech and noise stimuli in a free field, because it simulates situations which are similar to what we find in real communication situations.

Thus, based on these considerations and knowing that as we treat hearing disorders, it is necessary to first establish reference values obtained from audiologically normal individuals, in order to be able to understand the difficulties found by people with hearing disability, the following research project was created, aimed at determining reference values for speech recognition in noise thresholds, in free fields, for adult, normo-hearing individuals.

## MATERIALS AND METHODS

The present investigation was developed in the years of 2005 and 2006, after being registered at the Research Projects Department of our institution, where it was carried out under RP# 018269 and approved by the Ethics in Research Committee, under protocol # 051/2005.

The measures were obtained in a sound-treated booth, with a two-channel digital audiometer, from Fonix, model FA-12, type I; TDH- 39 P ear phones from Telephonics and an amplification system for free field audiometry. Phrases and noise were introduced through a Digital Toshiba CD player model 4149, coupled to the audiometer described above.

The procedures carried out were the following: Study Group selection

Only those individuals who agreed with the procedures necessary to perform the research and signed the Informed Consent Form, after having received more information on the study's objective and methodology.

The inclusion criteria we used were: Age between 18 and 64 years and audiometric thresholds within normal limits^9^.

As exclusion criteria we used the following: neurological, speech and/or verbal fluency alterations; ear wax or other alterations to the external acoustic meatus that can alter the test performance; no response to the Phrases in Portuguese List Test^8^ and difficulty to memorize phrases. We also excluded normo-hearing individuals who were unable to hear/understand properly or who had tinnitus.

In total, 150 individuals were selected, 70 men and 80 women.

### Audiologic evaluation

Initially, we had the patients answer a multiple-choice questionnaire about relevant personal information, educational level, profession, lifestyle, ear background and hearing complaints of the subjects studied. Such information was used to support the examiner during the test and to survey possible exclusion criteria, not for any posterior analysis.

Following that, we visually inspected the external acoustic meatus in order to remove from the sample those individuals with alterations capable of interfering in the results of the evaluations proposed.

Later on, the patients were submitted to basic audiologic evaluation, made up of: threshold tonal audiometry by air conduction in the frequencies of 250 to 8,000 Hz and bone conduction in the frequencies of 500 to 4,000 Hz; speech recognition threshold test, with dissyllable words that, according to the reference consulted^10^ must be used in order to obtain the threshold because they provide more reliable results; and also speech recognition percentage index. Both logoaudiometric tests were presented in ear phones and speaker mode.

### Obtaining phrases recognition thresholds (PRT)

After being submitted to basic audiologic evaluation, the selected individuals were assessed in order to obtain the phrases recognition threshold in noise (PRTN), in the free field, binaurally.

For such end, we applied the Portuguese Phrases List test^8^, made up of a list containing 25 phrases in Brazilian Portuguese, called List 1A^11^, seven lists with 10 phrases each, called 1B to 7B^12^ and a noise with speech spectrum^13^. The phrases and noise recorded in a CD, in independent channels, were presented through a CD player connected to the audiometer.

The test application in the free field was carried out in a sound-treated facility, with the individual placed at 1m from the sound source, in the following condition 0° – 0° azimuth, in other words, in front of the individual, without horizontal or vertical shifting^14^, since this is the condition that is closer to daily life communication situations^15^.

In order to answer the test, the individual had to repeat each phrase as he/she understood it; right after it was present to him/her.

We used different lists, one for each test condition, in order to eliminate the possibility of better performance due to memorizing the phrases. The use of different lists was not considered a variable, because the lists used in this study were equivalent^12^, ^13^, ^14^, ^15^, ^16^.

Although the equipment was previously calibrated according to technical standards, speech and noise stimuli were monitored during the entire investigation. In order to do that, we used a sound pressure level digital meter (SPLM) from Radio Shack, in order to establish and guarantee always the same free field acoustic conditions for all the patients under evaluation.

In order to calibrate the stimuli in the free field, we selected the A scale in the SPLM, with quick responses, which is more used to measure continuous noise and to determine extreme values of intermittent noise, and it is also the scale used by most researchers in this field.^3^^,^^5^^,^^15^^,^^17^, ^18^, ^19^.

In order to obtain the intensity levels found in the free field, we used the following strategy that has been already employed in prior investigations:^20^, ^21^

– Noise calibration: we adjusted the audiometer's VU meter in the 0 position and measured the noise SPL in the free field of the A scale of the SPLM. We observed a difference of 20 dB between the intensity recorded in the equipment's dial and the sound field measure. Such difference was added to the intensity shown in the dial, thus setting the noise intensity in the free field.
– Phrases calibration: considering the noise to be continuous, based on its intensity, we established the phrases presentation intensity. Prior studies 22, 23 observed that the phrases were recorded in a CD at an average intensity which was 7 dB below the noise intensity. Keeping such difference and knowing that, in the free field, the noise intensity was 20 dB above the value shown in the equipment's dial, the phrases presentation intensity was 13 dB above the one shown in the dial. For these measures, the audiometer's VU meter was put in the position 0 (zero).

For instance: when we noticed the value of 45 dB for the noise channel, it was presented in the free field at an intensity of 65 dB A. When we saw a value of 45 dB in the equipment's dial for the speech channel, the phrases were presented in the free field, at a real intensity of 58 dB H, result equal to −7 dB in a sound/noise ratio.

In assessing the individuals, we first familiarized them with the test. To do that, we presented them the phrases from 1 to 10 of list 1A, without the competitive noise. To allow assessed individuals to be able to answer correctly the first phrase from each list, and then, understand how the test worked, the initial presentation intensity of phrases in silence for the training list was 20 dBHL above the speech recognition threshold from the best ear.

Following that, we presented phrases 11 to 20 from list 1A, with competitive noise. In this case, the initial presentation intensity for this list was 63 dB H and the noise was at 65 dB A, and this makes an initial S/N ratio of −2.

In order to determine the phrases recognition threshold in noise for these individuals, we used list 1B, with competitive noise.

As it happened during the test familiarizing stage, the initial S/N for the list presentation was of −2.

The strategy used to investigate the speech recognition threshold in noise was the sequential or adaptative, or even the ascending-descending, proposed by LEVIT & RABINER (1967). This one allows for a necessary level for the individual to correctly identify approximately 50% of the speech stimuli presented in an established S/N ratio. Although the suggestion from these authors was to use 4 dB intervals until the first change in response type and, later on, the stimuli presentation intervals be of 2 dB among each other until the end of the list, because of the technical possibilities of the equipment available for this research project, we used phrases' presentation intervals of 5 dB and 2.5 dB, respectively.

In this procedure, we presented a stimulus at a given S/N ration. If the individual correctly identified the speech stimulus presented, its intensity was reduced in pre-established intervals, and if not, the intensity was increased.

This procedure was repeated until the end of the list.

In order to obtain sound recognition thresholds in silence (SRTS), used for training, the phrases' presentation levels were noted in order to later calculate an average based on the values when there was a change in response type. It is important to stress that the results obtained from the training of the individuals were not considered in the statistical analysis.

To obtain the SRTN, the procedure was the same, the value obtained was subtracted from the noise level presented (65 dB H), thus obtaining the S/N ratio at which the individual was able to recognize about 50% of the stimuli presented.

### Statistical Analysis

In order to present a summarized study of the results obtained from this investigation, our basis for data description was the calculation of standard deviation average values and the maximum and minimum points attained in the SRTN evaluation.

## RESULTS

Table 1 shows the descriptive statistical analysis for the phrases recognition thresholds in noise data for the 150 normo-hearing individuals evaluated.

**Table 1:** Descriptive statistical analysis of phrases recognition thresholds in noise for normo-hearing individuals.

N	AVERAGE	MAXIMUM	MINIMUM	STANDARD DEVIATION
150	−8.14	−4.77	−13.07	1.69

As we can see, the average S/N ratio at which we obtained the PRTN for the individuals assessed was of −8.14 dB A.

In the literature studied, we found the values presented in Chart 1 from similar studies.

**Chart 1:** PRTN obtained from normo-hearing individuals, according to the authors studied.

AUTOR	PRTN (dBH)
Bronkhorst & Plomp15	−6.4
Gelfand et al.24	−2
Nilsson et al.19	−2.60
Costa et al.11	−10.33
Kramer et al.25	−12
Ribeiro26	−6
Pagnossim et al.27	−6.71
Miranda & Costa20	−8.72
Henriques & Costa21	−7.56
Henriques28	−7.57

We notice that the results obtained by these researchers vary broadly among themselves, which makes it difficult to reach a consensus and establish a standard value, despite the fact that most of these studies followed similar methodology. This variation can be justified for a number of aspects that can impact these measures and must be considered in order to have a more reliable interpretation of the findings.

Initially, we can mention the variables found in speech tests performed in free fields as: room size, acoustic conditions, whether or not there is a reflective surface, level of reverberation, calibration and even the number of people inside the test facility.

We also stress that some tests were developed in different languages, thus, linguistic factors such as speech experience and language mastering can impact the results^29^.

Thus, the values presented serve as a reference for studies that follow the same test conditions. Nonetheless, we suggest that each audiologist establish his/her own parameters considering the situation at which the patients are being assessed.

It is important to notice that the value of −8.14 dB A is different from the one considered as reference for the assessment with ear phones^16^, which is −5.29 dB. This is so because, although the test and its application strategy are the same, the presentation in the free field suffers influence from the acoustic conditions of the test facilities, which are totally eliminated when the stimuli are presented by means of ear phones. In this way, the professional that applies the test should not compare the values obtained through the ear phones to data collected in the free field and vice-versa in order to avoid mistakes in finishing the evaluations.

## CONCLUSION

A thorough data analysis allowed us to conclude that the reference value for the phrases recognition thresholds in noise using the LSP test in free field for normo-hearing individuals is −8.14 dBH.
